# Real-world outcomes after switching from standard therapy to efgartigimod in five patients with chronic inflammatory demyelinating polyradiculoneuropathy: a case series study in Japan

**DOI:** 10.3389/fneur.2026.1748826

**Published:** 2026-03-13

**Authors:** Tatsuya Imai, Kenichi Irie, Shinichiro Mori, Tomoaki Hoshino, Toshihiro Ide, Takahisa Tateishi

**Affiliations:** 1 Division of Respirology, Neurology and Rheumatology, Department of Medicine, Kurume University School of Medicine, Kurume, Japan; 2 Department of Neurology, Saga University, Saga, Japan

**Keywords:** case series study, chronic inflammatory demyelinating polyradiculoneuropathy, CIDP variant, efgartigimod, Japan, neonatal Fc receptor blocker, typical CIDP

## Abstract

**Background:**

Efgartigimod, a neonatal Fc receptor blocker, has received regulatory approval for the treatment of chronic inflammatory demyelinating polyradiculoneuropathy (CIDP) in Japan in December 2024.

**Aims:**

To investigate the effectiveness and safety of efgartigimod in real-world clinical setting immediately after its approval.

**Methods:**

We conducted a prospective, single-center, case series study to evaluate the switch from standard therapy to efgartigimod in five patients with typical or variant CIDP in Japan. Effectiveness was assessed with clinical responses defined by changes in the Inflammatory Rasch-built Overall Disability Scale, Inflammatory Neuropathy Cause and Treatment, Medical Research Council sum scores, and grip strength. The occurrence of adverse events (AEs) was also monitored.

**Results:**

Three patients had typical CIDP, one had variant CIDP of the motor type, and one had distal type CIDP. All patients exhibited a clinical response evaluated using at least one effectiveness endpoint after switching to efgartigimod treatment. Especially, the three patients with typical CIDP experienced an significant effectiveness of efgartigimod, even in those with an inadequate response to intravenous or subcutaneous immunoglobulin. Conversely, one patient with distal CIDP did not exhibit a response to efgartigimod treatment. One patient experienced a severe headache after efgartigimod treatment; however, the AE was manageable.

**Conclusion:**

Efgartigimod is a useful treatment option for CIDP in real-world clinical practice. However, its effectiveness was different between the patients with typical CIDP and CIDP variant in our study, possibly due to variances in the immune pathophysiology of each disease subtype. Further validation is warranted in our exploratory findings.

## 1 Introduction

Chronic inflammatory demyelinating polyradiculoneuropathy (CIDP) is an autoimmune disorder of the peripheral nervous system characterized by progressive or relapsing muscle weakness and sensory disturbances lasting for at least 8 weeks (1–3). CIDP is classified into several subtypes: typical CIDP is the most common form, presenting symmetrical involvement and proximal as well as distal muscle weakness. CIDP variants such as distal, multifocal, focal, motor, and sensory CIDP have different clinical presentations from typical CIDP, although they share common features of demyelination and response to immune therapy (1–3). Patients with CIDP experience physical and psychosocial burdens, including impaired physical function, pain, and depression, which may lead to impaired quality of life (QOL) (4).

First-line therapies for CIDP are steroids, immunoglobulins, and plasma exchange, which have proven effective in inducing and maintaining remission. However, these therapies are rarely associated with complete resolution or cure and often entail significant practical, financial, and medical challenges (5). New therapies for CIDP, which pose different immunopathological mechanism of actions from the first-line therapies, are currently approved and in the developmental stage. These therapies target the cellular, humoral, and complement pathways that play a crucial role in peripheral nerve damage in CIDP (6). The neonatal Fc receptor (FcRn) plays a pivotal role in prolonging the serum half-life of IgG (7). FcRn blockade to prevent its binding to IgG can accelerate the catabolism of IgG, resulting in decreased levels of IgG, thereby achieving a therapeutic effect on the pathogenic IgG-related disorders (8). Efgartigimod, a FcRn blocker, has demonstrated a reduced risk of relapse with observed improvements in disability, strength, and QOL scores in patients with CIDP, demonstrating favorable tolerability in the pivotal, global phase 2 ADHERE trial (9). Based on these findings, efgartigimod received regulatory approval for the treatment of CIDP in the US in June 2024, and in Japan in December 2024.

There has been limited evidence that assess the effectiveness and safety of efgartigimod in real-world clinical setting after its approval, although some case series studies have indicated these results (10, 11). Here, we report the findings that evaluate the effectiveness and safety of efgartigimod in five patients with CIDP in clinical practice in Japan.

## 2 Materials and methods

### 2.1 Study design

This prospective, single-center, case series study aimed to evaluate the effectiveness and safety of efgartigimod in patients with CIDP in real-world clinical practice in Japan. The study was conducted from December 2024 to July 2025 in accordance with the Declaration of Helsinki ethical guidelines. All participated patients provided written informed consent before entering the study.

### 2.2 Patients

Study patients were five individuals who were clinically and electrophysiologically diagnosed with CIDP according to the 2021 European Academy of Neurology and Peripheral Nerve Society criteria (1), and their therapy was switched from standard therapy to efgartigimod between the period of December 2024 to April 2025.

### 2.3 Treatment

All five patients received subcutaneous efgartigimod once weekly at a dosage of 1,008 mg according to the package insets of the drug (12).

### 2.4 Study assessments

Effectiveness endpoints included changes in disability scales such as the Inflammatory Rasch-built Overall Disability Scale (IRODS) and Inflammatory Neuropathy Cause and Treatment (INCAT) scores, and impairment scales, such as the Medical Research Council (MRC) sum score and grip strength. A clinical response in each effectiveness endpoint was defined as meeting *a* ≥ 4 points increase in the IRODS score, *a* ≥ 1 points decrease in the INCAT score, *a* ≥ 4 points increase in the MRC sum score, or *a* ≥ 10% increase in grip strength. Effectiveness of efgartigimod was defined by the fulfillment of ≥1 endpoint of both disability and impairment scales after the initiation of efgartigimod administration according to previous guidance (1, 13). The safety assessment endpoint was the incidence of adverse events (AEs) after efgartigimod treatment. Serum IgG and albumin values were also measured before and after efgartigimod treatment. Effectiveness and safety were evaluated in five patients with CIDP at their hospital visits before and after the initiation of efgartigimod administration. The baseline scores were taken immediately before the first efgartigimod administration. The evaluation was continued for up to 12 weeks after the initiation of efgartigimod administration.

### 2.5 Statistical analysis

We descriptively analyzed the data, and no statistical hypothesis tests were conducted.

## 3 Results

**Figure 1:**
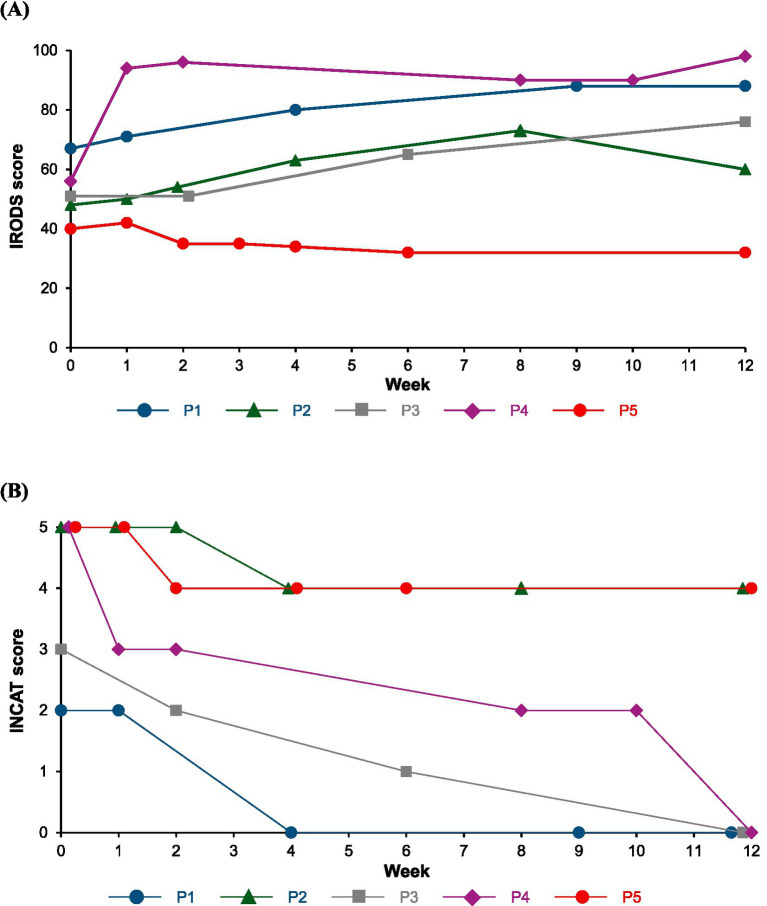
Change in disability scales of the IRODS **(A)** and INCAT **(B)** scores. P1: Patient 1, P2: Patient 2, P3: Patient 3, P4: Patient 4, P5: Patient 5. INCAT, Inflammatory Neuropathy Cause and Treatment; IRODS, Inflammatory Rasch-built Overall Disability Scale.

**Figure 2:**
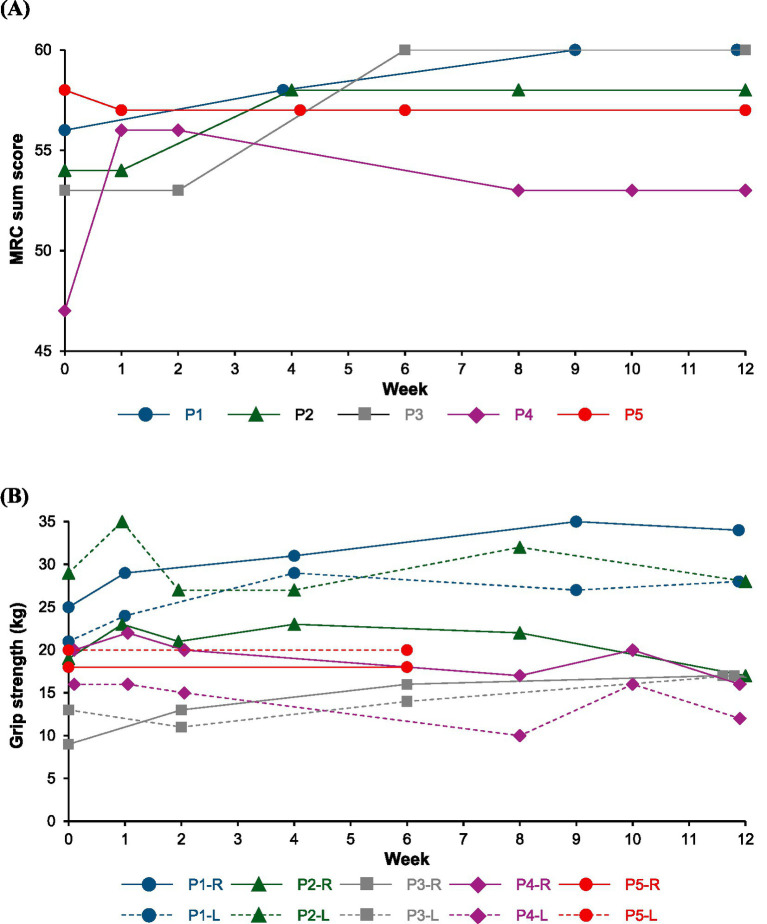
Change in impairment scales of the MRC sum score **(A)** and grip strength **(B)**. P1: Patient 1, P2: Patient 2, P3: Patient 3, P4: Patient 4, P5: Patient 5, R, right hand; L, left hand; MRC, Medical Research Council.

**Figure 3:**
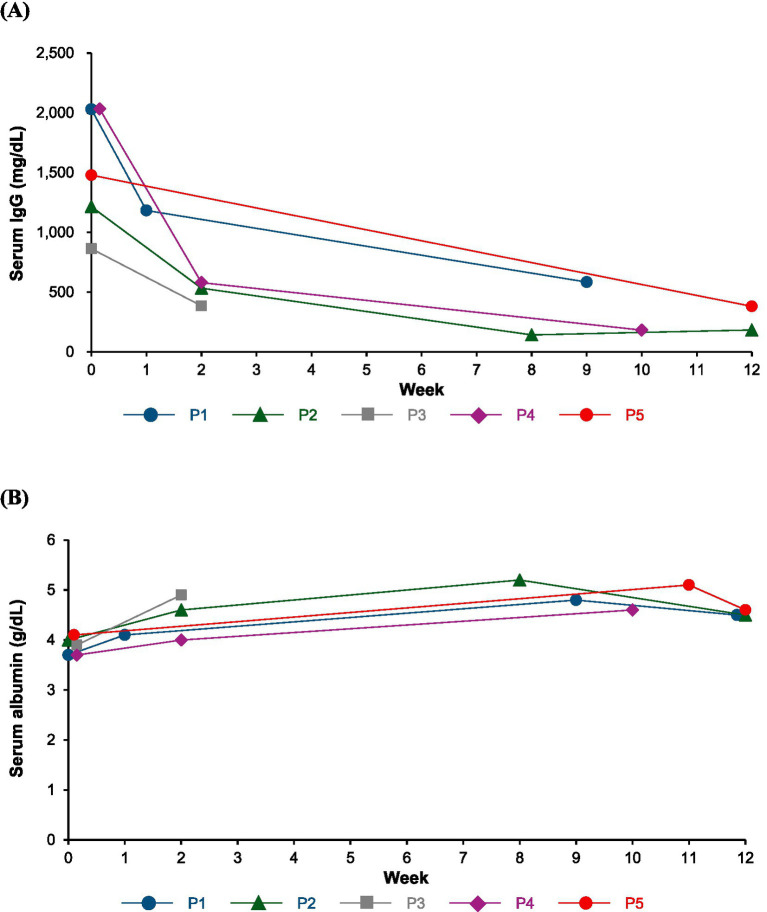
Change in serum IgG **(A)** and albumin **(B)** values. P1: Patient 1; P2: Patient 2; P3: Patient 3; P4: Patient 4; P5: Patient 5.

Patient characteristics is shown in Table 1, and changes in the IRODS, INCAT, MRC sum score, grip strength, serum IgG and albumin values after the initiation of efgartigimod administration are described in Figures 1–3.

**Table 1:** Patient characteristics.

Characteristics	Patient 1	Patient 2	Patient 3	Patient 4	Patient 5
Age, years	50	65	53	22	36
Sex	Male	Male	Male	Female	Male
Time from onset to diagnosis, month	2	29	20	2	90
Onset type	Chronic	Chronic	Chronic	Chronic	Chronic
CIDP type	Typical	Typical	Typical	Variant (motor)	Variant (distal)
Muscle weakness site	UL + LL	UL + LL	UL + LL	UL + LL	UL + LL
Sensory impairment site	UL + LL	UL + LL	UL + LL	None	UL + LL
Muscle atrophy site	None	None	None	UL	LL
Electrophysiological examination	Sensorimotor demyelinating polyneuropathy	Sensorimotor demyelinating polyneuropathy	Sensorimotor demyelinating polyneuropathy	Motor demyelinating polyneuropathy	Sensorimotor demyelinating polyneuropathy
CSF protein level, mg/dL (reference range: 8–43 mg/dL)	85	82	111	22	28
Comorbidity	None	None	None	None	None
Response to prior therapy					
IVIg	Effective	Partially effective	Partially effective	Effective	Ineffective
SCIg	Partially effective	Partially effective	Not used	Partially effective	Not used
Plasma exchange	Not used	Not used	Not used	Not used	Ineffective
Corticosteroids	Ineffective	Partially effective	Partially effective	Partially effective	Not used
Time from diagnosis to EFG administration, month	13	9	72	7	29
Therapy immediately prior to EFG administration (period until switching to EFG administration)	IVIg (6 weeks)	IVIg (4 weeks)	IVIg (4 weeks)	SCIg (2 weeks)	IVIg (4 weeks)
Concomitant medications with EFG administration	Not used	PSL, tacrolimus	PSL, azathioprine	PSL	Not used
PSL oral dosage (prior to EFG administration/at final follow-up)	Not used	12.5 mg/7.5 mg	10 mg/2.5 mg	10 mg/5 mg	Not used

CIDP, chronic inflammatory demyelinating polyradiculoneuropathy; CSF, cerebrospinal fluid; EFG, efgartigimod; IVIg, intravenous immunoglobulin; LL, lower limbs; PSL, prednisolone; SCIg, subcutaneous immunoglobulin; UL, upper limbs.

### 3.1 Patient 1 (50-year-old man)

After a diagnosis of typical CIDP, he received intravenous immunoglobulin (IVIg) every 4 weeks and continued treatment during his hospital visits due to its effectiveness. The treatment was switched to self-injectable subcutaneous immunoglobulin (SCIg) because of his work commitments; however, muscle weakness gradually progressed after the switch. He received an additional treatment with 10 mg of oral prednisolone (PSL), but the symptoms did not improve. PSL treatment was discontinued, and the treatment was switched to IVIg again. Although the IVIg treatment was effective, muscle weakness was observed 2–3 weeks after treatment initiation. At 13 months post-diagnosis, the treatment was switched to efgartigimod considering the ability to self-administer at home and its weekly dosing. Muscle strength improved rapidly, with the MRC score increase to 60 points after 9 weeks of the switching, although mild abnormal sensations in the upper limbs persisted. He subjectively noted the treatment’s effects as early as one week after efgartigimod administration, and at 12 weeks after the treatment, the symptoms remained stable without fluctuations which had been previously observed.

### 3.2 Patient 2 (65-year-old man)

After the diagnosis of typical CIDP, he received IVIg and treatment was switched to SCIg. One week after switching, muscle weakness gradually progressed, and he received additional treatment with 10–15 mg oral PSL, but the effectiveness was insufficient. Treatment was switched to IVIg again with PSL administration. No progression of muscle weakness was observed; however, fluctuations occurred around 3 weeks after IVIg treatment initiation. Additional tacrolimus was administered, but the symptoms did not improve. At 9 months post-diagnosis, treatment was switched to efgartigimod with PSL and tacrolimus administration. After 1 week of switching, muscle strength tended to improve, with a slight increase in the IRODS score, and improvements in the INCAT and MRC scores were also observed after 4 weeks of treatment. The PSL dosage was reduced to 7.5 mg, while tacrolimus treatment was continued. Although the IRODS score slightly decreased, possibly due to the reduction in PSL dosage, it remained higher than the baseline value, and improvements in the INCAT and MRC scores continued for 12 weeks after treatment.

### 3.3 Patient 3 (53-year-old man)

After the diagnosis of typical CIDP, he was treated with 10 mg oral PSL along with 50 mg azathioprine, and his condition remained stable. Three years after the diagnosis, muscle weakness progressed again, and he received additional treatment with IVIg every 4 weeks, with no disease progression. However, muscle weakness and fluctuations occurred 2–3 weeks after the IVIg treatment initiation. At 72 months post-diagnosis, the patient transitioned to efgartigimod from IVIg. Muscle strength improved rapidly, with the MRC score increasing to 60 points after 6 weeks of switching, and no fluctuations were observed. Twelve weeks after the treatment, the symptoms remained stable, and the PSL dosage was reduced.

### 3.4 Patient 4 (22-year-old woman)

After a diagnosis of variant CIDP of motor type, she underwent treatment with SCIg and oral PSL for 2 weeks, gradually tapering the PSL dosage. The treatment was discontinued due to fluctuations and severe headaches, leading to a switch from SCIg to efgartigimod at 7 months post-diagnosis. Muscle strength improved after switching, with no fluctuations; however, severe headaches occurred after each efgartigimod administration, which persisted for several days even when treated with intravenous PSL or acetaminophen. The headache severeness got better, although not completely resolved, by slowly injecting efgartigimod subcutaneously over 5 min, allowing treatment continuation. After 8 weeks treatment, the IRODS score was reduced because of cervical sprain caused from a traffic accident, activities of daily living improved compared with that prior to efgartigimod treatment. Additionally, she required a wheelchair and caregiver for her hospital visit before the treatment initiation; however, the disease improved, and she was able to walk independently with braces after 2 weeks treatment, and visit hospital without braces and caregiver after 8 weeks treatment.

### 3.5 Patient 5 (35-year-old man)

After a diagnosis of variant CIDP of distal type, he received IVIg treatment, but the effectiveness was insufficient, with no subjective symptom improvement. IVIg treatment was discontinued, and he was followed-up without any treatments, including steroids and immunosuppressants administrations due to his request. About one year before the efgartigimod treatment initiation, the disease progressed, and IVIg was administered again, but the effectiveness was insufficient. IVIg was switched to efgartigimod at 29 months post-diagnosis. After the first week of efgartigimod treatment, slight symptom improvement was observed; however, the disease progressed thereafter. A nerve biopsy revealed a pathological examination of severe axonal damage, without findings of other suspectable diseases such as amyloid polyneuropathy (detailed histopathological findings are provided in Supplementary Figures S1–S3). M protein was negative and we determined that monoclonal gammopathy could be ruled out as a diagnosis. Nerve ultrasonography and magnetic resonance neurography (MRN) detected as enlargement of nerve roots, and electrophysiological test showed a long-term deterioration of demyelinating features. These findings suggested the development of secondary axonal damage.

### 3.6 Effectiveness

In this case series of five patients with CIDP, all patients showed a clinical response evaluated by at least one effectiveness endpoint after switching from standard therapy to efgartigimod treatment. Two patients (Patient 1 and 3) exhibited clinical improvement across all effectiveness endpoints. Effectiveness of efgartigimod was achieved in four patients (Patient 1–4), and the effectiveness was observed as early as one week after efgartigimod treatment in some patients, and by 6 weeks after the treatment in all four patients. Patient 5, who had variant CIDP of distal type and the longest disease duration of 119 months, exhibited only a 1-point improvement in the INCAT score, with no improvement in other effectiveness endpoints.

### 3.7 Safety

AE occurred only in Patient 4, who experienced severe headaches after efgartigimod treatment. The severity of the headache improved by slowly injecting efgartigimod subcutaneously over 5 min, and the patient was able to continue the treatment. No AEs, including infections, were observed in the other four patients. A decrease in serum IgG values were observed in all patients, while albumin values were slightly increased after efgartigimod treatment.

## 4 Discussion

In this real-world case series, four of five patients with CIDP demonstrated clinical effectiveness after switching from standard therapy to efgartigimod administration in five patients with CIDP, four patients, including all three patients with typical CIDP. Among them, two patients exhibited marked improvement across all evaluated endpoints after 12 weeks of treatment. These findings are broadly consistent with the pivotal global phase 2 ADHERE trial (9), in which 214 of 322 patients (66%) achieved evidence of clinical improvement—defined as *a* ≥ 1-point adjusted INCAT decrease, *a* ≥ 4-point IRODS increase, or *a* ≥ 8-kPa grip strength increase—after up to 12 weeks of efgartigimod administration. Notably, 83% of participants in ADHERE had typical CIDP. Although direct comparison between our study and ADHERE is limited by differences in patient characteristics, outcome definitions, and study design, the overall concordance suggests that efgartigimod is effective for typical CIDP in routine clinical practice.

Two patients with CIDP variants were included in our series: one with motor CIDP and one with distal CIDP. The motor CIDP patient responded to efgartigimod, whereas the distal CIDP patient did not. CIDP variants differ substantially in clinical presentation, pathology, electrophysiology, and responsiveness to standard therapies that are effective in typical CIDP (14). Evidence regarding the efficacy of efgartigimod in CIDP variants remains limited. A previous case series reported that a patient with motor CIDP and relatively mild symptoms (disease duration of 14 months) achieved clinical improvement—defined as *a* ≥ 1-point INCAT decrease—by week 4 after two weekly doses of efgartigimod, with parallel improvements in IRODS, MRC sum scores, and grip strength (11). In our study, the motor CIDP patient initiated efgartigimod 7 months after diagnosis and also responded favorably. Together, these observations suggest that early introduction of efgartigimod may be beneficial in motor CIDP, although confirmation in larger cohorts is required.

In contrast, the patient with distal CIDP—who had the longest disease duration (119 months)—showed only a minimal 1-point INCAT improvement and no gains in other endpoints. Multimodal evaluation, including nerve biopsy, ultrasonography, MRN, and electrophysiology, revealed severe secondary axonal damage. CIDP is known to cause secondary axonal degeneration in addition to demyelination; early disease stages may involve reversible conduction block, but chronic demyelination and remyelination eventually lead to Wallerian degeneration and axonal loss. Iijima et al. (15) further demonstrated that muscle atrophy and reduced compound muscle action potentials are more pronounced in IVIg non-responders, implicating axonal dysfunction in treatment resistance. These findings suggest that therapies primarily targeting antibody removal may be less effective once substantial axonal damage has occurred. Efgartigimod accelerates IgG catabolism via FcRn blockade (8), and may therefore be insufficient in patients whose disability is driven predominantly by irreversible axonal loss. A previous case series similarly reported clinical deterioration after switching from IVIg to efgartigimod in a distal CIDP patient with a long disease duration (84 months) (10), supporting our observation. Collectively, these data imply that FcRn blockade may be most effective when initiated before significant axonal degeneration develops, whereas corticosteroid-based therapy may be more appropriate in advanced axonal pathology.

Treatment-related fluctuations (TRFs) are frequently observed in patients receiving cyclic IVIg, typically emerging 2–3 weeks after infusion (16). These fluctuations may reflect transient neutralization of pathogenic IgG and waning immunomodulatory effects over time (17). In our series, four patients experienced recurrent TRFs during IVIg therapy. After switching to efgartigimod, three of these patients achieved stable improvement without further fluctuations. Efgartigimod has been shown to reduce relapse risk for up to 48 weeks in CIDP (9), suggesting that sustained IgG reduction may mitigate symptom variability.

Pharmacokinetic studies demonstrate that IVIg produces high peak IgG levels followed by rapid declines, whereas SCIg provides slower absorption and more stable trough concentrations (18, 19). Real-world data indicate that switching from IVIg to SCIg can reduce functional fluctuations, likely due to reduced variability in serum IgG levels (20). Comparative studies further show that SCIg maintains higher trough IgG levels than IVIg despite equivalent monthly dosing, highlighting the importance of dosing interval and absorption kinetics (21). In our study, three patients transitioned from IVIg to SCIg but continued to experience TRFs. Importantly, these patients were receiving relatively low SCIg doses, and escalation to optimized high-dose SCIg was not fully pursued due to clinical or practical considerations. Thus, the limited efficacy of SCIg in our cohort may reflect the timing of the switch rather than true therapeutic insufficiency, as individualized dose optimization is often required for stable disease control.

Nevertheless, persistent TRFs or insufficient improvement despite IVIg or low-dose SCIg prompted initiate of efgartigimod based on clinical judgment and patient-centered considerations. Although Cook et al. (22) reported that TRFs during IVIg therapy do not reliably predict long-term disease activity, such fluctuations can substantially impair quality of life, warranting reconsideration of treatment strategy when clinically burdensome. Unlike the variable IgG exposure associated with IVIg or low-dose SCIg, weekly subcutaneous efgartigimod may suppress fluctuations by achieving more consistent IgG reduction. Future studies should clarify the relative benefits, optimal sequencing, and cost-effectiveness of immunoglobulin optimization versus FcRn blockade in CIDP management.

All patients in this study successfully self-administered subcutaneous efgartigimod at home. Although clinical improvement remains the primary therapeutic goal, patient satisfaction was notably high due to the increased flexibility and autonomy. While no formal QOL scale was used, all four responders reported subjective improvements related to treatment convenience and self-management. These observations are consistent with prior reports of home-based immunoglobulin therapy. Markvardsen et al. (23) found that many patients who transitioned from IVIg to SCIg preferred the latter, citing improved stability of muscle strength, fewer adverse effects, and greater time efficiency; enhanced autonomy and convenience were the most frequently cited advantages. Incorporating patient-centered perspectives is therefore essential when designing individualized CIDP treatment strategies.

Regarding safety, one patient experienced a severe headache after efgartigimod administration. This adverse event was manageable by slowing the subcutaneous injection to 5 min and administering headache medication, allowing treatment continuation. In the phase 2 trial, headache occurred in 5% of patients receiving subcutaneous efgartigimod, and overall tolerability was favorable (9). Although efgartigimod reduces IgG levels and may theoretically increase infection risk, no infections occurred in our cohort. FcRn also recycles albumin, but albumin and IgG bind to distinct sites, allowing concurrent interaction (24). Consistent with phase 1 data, no reductions in serum albumin were observed in our study (25).

## 5 Conclusion

In this real-world case series study of five patients with treatment-resistant CIDP, four achieved a clinical response defined using changes in the IRODS, INCAT, MRC sum score, and grip strength after efgartigimod administration. Particularly, patients with typical CIDP experienced significant effectiveness of efgartigimod; even in patients with an inadequate response to IVIg or SCIg, efgartigimod demonstrated stable improvement and contributed to the reduction of fluctuations. Overall, the observed reduction in TRF and increased flexibility provided by home administration enhanced the QOL in patients with CIDP. In contrast, one case of the CIDP subtype (distal CIDP) showed resistance to efgartigimod treatment and exhibited marked axonal damage. This observation suggests that disease duration, disability of severity, and differences in the immune pathophysiology of each disease subtype may impact treatment effectiveness. Efgartigimod is a useful treatment option for CIDP in real-world clinical practice; however, further validation is warranted in this setting.

## Data Availability

The original contributions presented in the study are included in the article/Supplementary material, further inquiries can be directed to the corresponding author.
