# Supplementary material for: Real-world outcomes after switching from standard therapy to efgartigimod in five patients with chronic inflammatory demyelinating polyradiculoneuropathy: a case series study in Japan
Source: Front Neurol. 2026 Mar 13;17:1748826. doi: 10.3389/fneur.2026.1748826 (PMC13023133; doi:10.3389/fneur.2026.1748826)
Supplement: Supplementary file 1 [file presentation_1.pptx]

## Slide 1
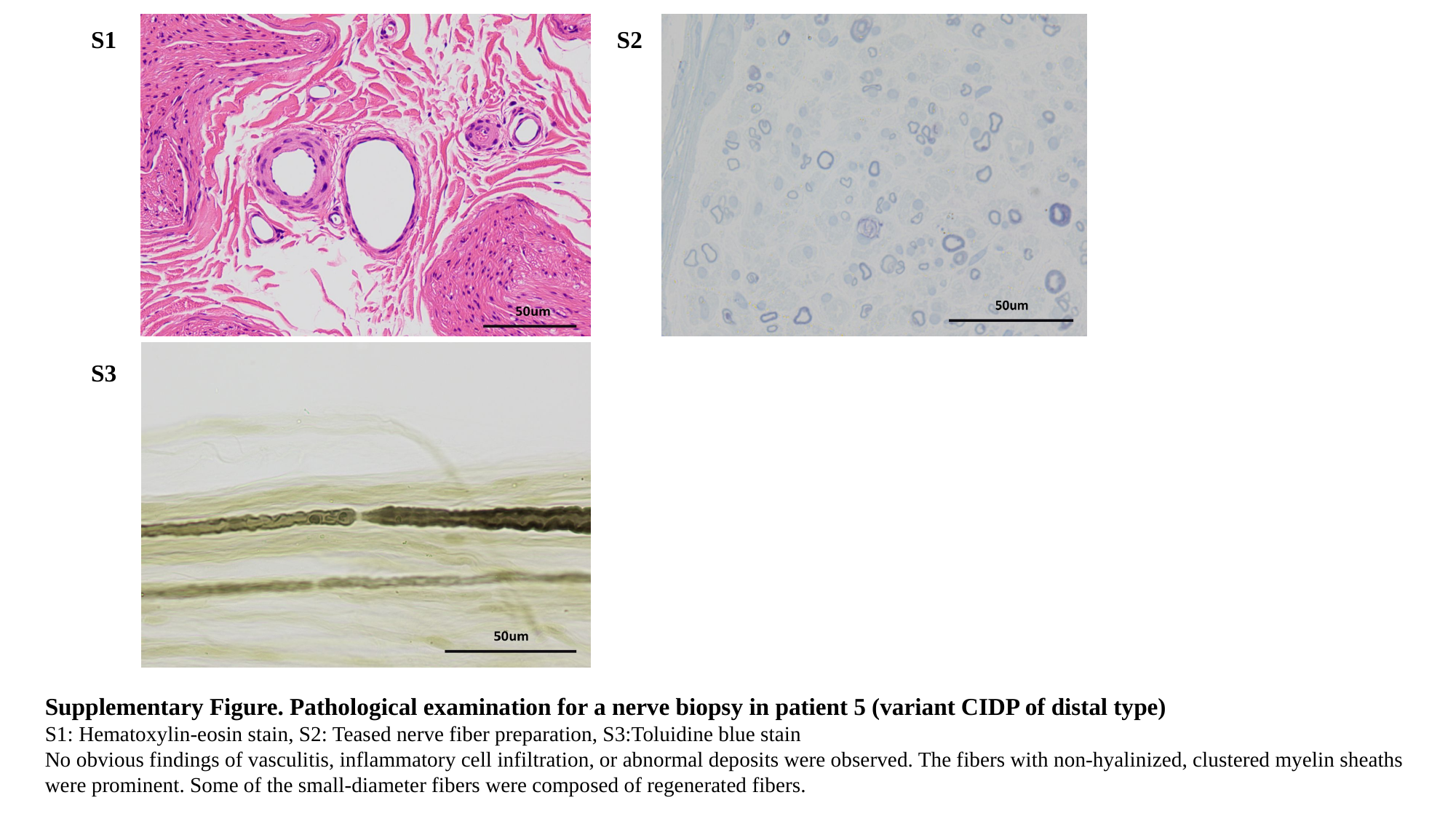

S1
S2
S3
Supplementary Figure. Pathological examination for a nerve biopsy in patient 5 (variant CIDP of distal type)
S1: Hematoxylin-eosin stain, S2: Teased nerve fiber preparation, S3:Toluidine blue stain
No obvious findings of vasculitis, inflammatory cell infiltration, or abnormal deposits were observed. The fibers with non-hyalinized, clustered myelin sheaths were prominent. Some of the small-diameter fibers were composed of regenerated fibers.
